# Preactivation of Notch1 in remote ischemic preconditioning reduces cerebral ischemia-reperfusion injury through crosstalk with the NF-κB pathway

**DOI:** 10.1186/s12974-019-1570-9

**Published:** 2019-09-16

**Authors:** Weidong Liang, Chunshui Lin, Liuqing Yuan, Li Chen, Peipei Guo, Ping Li, Wei Wang, Xin Zhang

**Affiliations:** 1 Nanfang Hospital, Southern Medical University, Guangzhou, 510515 China; 2 The First Affiliated Hospital of Gannan Medical University, Ganzhou, 341000 China; 3 Department of Anesthesia, Guangdong Provincial Hospital of Chinese Medicine, Guangzhou, 510120 China

**Keywords:** Ischemia-reperfusion injury, Remote ischemic preconditioning, Neuroprotection, Notch1 pathway, NF-kappa B, Cross reaction

## Abstract

**Background:**

Remote ischemic preconditioning (RIPC) initiates endogenous protective pathways in the brain from a distance and represents a new, promising paradigm in neuroprotection against cerebral ischemia-reperfusion (I/R) injury. However, the underlying mechanism of RIPC-mediated cerebral ischemia tolerance is complicated and not well understood. We reported previously that preactivation of Notch1 mediated the neuroprotective effects of cerebral ischemic preconditioning in rats subjected to cerebral I/R injury. The present study seeks to further explore the role of crosstalk between the Notch1 and NF-κB signaling pathways in the process of RIPC-induced neuroprotection.

**Methods:**

Middle cerebral artery occlusion and reperfusion (MCAO/R) in adult male rats and oxygen-glucose deprivation and reoxygenation (OGD/R) in primary hippocampal neurons were used as models of I/R injury in vivo and in vitro, respectively. RIPC was induced by a 3-day procedure with 4 cycles of 5 min of left hind limb ischemia followed by 5 min of reperfusion each day before MCAO/R. Intracerebroventricular DAPT injection and sh-Notch1 lentivirus interference were used to inhibit the Notch1 signaling pathway in vivo and in vitro, respectively. After 24 h of reperfusion, neurological deficit scores, infarct volume, neuronal apoptosis, and cell viability were assessed. The protein expression levels of NICD, Hes1, Phospho-IKKα/β (p-IKK α/β), Phospho-NF-κB p65 (p-NF-κB p65), Bcl-2, and Bax were assessed by Western blotting.

**Results:**

RIPC significantly improved neurological scores and reduced infarct volume and neuronal apoptosis in rats subjected to I/R injury. OGD preconditioning significantly reduced neuronal apoptosis and improved cell viability after I/R injury on days 3 and 7 after OGD/R. However, the neuroprotective effect was reversed by DAPT in vivo and attenuated by Notch1-RNAi in vitro. RIPC significantly upregulated the expression of proteins related to the Notch1 and NF-κB pathways. NF-κB signaling pathway activity was suppressed by a Notch1 signaling pathway inhibitor and Notch1-RNAi.

**Conclusions:**

The neuroprotective effect of RIPC against cerebral I/R injury was associated with preactivation of the Notch1 and NF-κB pathways in neurons. The NF-κB pathway is a downstream target of the Notch1 pathway in RIPC and helps protect focal cerebral I/R injury.

## Introduction

Stroke remains a leading cause of death and disability worldwide, and few clinically approved treatments are available for its most common form, ischemic stroke. Ischemic preconditioning (IPC) is considered an effective prophylactic treatment for ischemic stroke. Many basic and clinical studies have confirmed that IPC can produce a significant neuroprotective effect by initiating endogenous protective mechanisms, effectively reducing cerebral ischemia-reperfusion injury [1–3]. Among the many ischemic preconditioning methods, remote ischemic preconditioning (RIPC) is considered a promising one, because it uses a simple, noninvasive procedure and is nearly ready for clinical practice. Numerous clinical trials in recent years have shown that RIPC can effectively induce cerebral ischemic tolerance, thereby reducing ischemia-reperfusion injury and improving patient prognosis. However, the underlying mechanism is not fully understood. Understanding the mechanisms underlying RIPC-induced neuroprotection will help minimize ischemic brain injury.

Evidence suggests that the protective effects of RIPC against ischemia are mainly related to neuronal, humoral, and immunity-related pathways, as well as a variety of biomolecules and signaling pathways. The Notch signaling pathway is a highly conserved pathway widely found in vertebrates and nonvertebrates, regulating the differentiation and development of cells, tissues, and organs through interactions with adjacent cells; the pathway plays an important regulatory role in cell proliferation, differentiation, and apoptosis. Previous studies suggest that the Notch signaling pathway is involved in cerebral ischemic injury, and blocking Notch signaling can reduce neuronal cell apoptosis and improve prognosis [4]. However, recent studies have confirmed that Notch signaling preactivation may be involved in neuronal ischemic tolerance to subsequent lethal ischemia-reperfusion injury [5–7]. It was reported that pretreatment with sevoflurane and isoflurane could reduce neuronal cell apoptosis during ischemia-reperfusion injury and promote proliferation of neural stem cells by activating the Notch signaling pathway; the protective effect of pretreatment was reversed if Notch signaling was blocked [5, 8]. Zhou et al. also demonstrated that ischemic preconditioning could reduce myocardial ischemia and reperfusion injury by activating the Notch signaling pathway [9]. Recent research confirmed that RIPC increased Notch signaling activity and promoted arteriogenesis in the ischemic rat brain [10].

Evidence confirms that NF-κB plays an important role in the formation of cerebral ischemic tolerance by regulating the transcriptional expression of target genes and the activity of other pathways involved in cerebral ischemia. Ischemic preconditioning can activate NF-κB signaling, increase the expression of NF-κB, and enhance the DNA-binding activity of that protein in neurons, thus inducing ischemic tolerance to subsequent lethal ischemia and hypoxia injury. Moreover, the NF-κB inhibitor PDTC can reverse the neuroprotective effect of IPC [11, 12]. Studies have shown that the gene NF-κB is a downstream target of Notch signaling pathway. The Notch signaling pathway regulates the activation of DNA binding of NF-κB by regulating IκB kinase (IKK), thus affecting the expression of NF-κB-dependent target genes [13, 14]. Additionally, the Notch ligand Jagged-1 is regulated by NF-κB [15]. A certain level of Notch signaling is essential to maintain the activity of NF-κB, and the activity of NF-κB decreases with the reduction of Notch signal intensity, showing a synergistic trend [16–18].

Accordingly, the aim of this study was to investigate the neuroprotective effects of RIPC on cerebral ischemia-reperfusion injury and the roles of the Notch1 and NF-κB signaling pathways in RIPC-induced neuroprotection.

## Methods

### Animals

Adult male Sprague Dawley rats (250–280 g, Laboratory Animal Center of Southern Medical University, Guangzhou, China) were housed with free access to standardized chow and tap water under diurnal lighting conditions (12-h/12-h light/dark cycle). All experimental protocols and animal-related procedures were carried out in accordance with the US National Institutes of Health’s Guide for the Care and Use of Laboratory Animals (NIH Publication No. 85-23, revised 1996) and approved by the Experimental Animal Ethics Committee of Southern Medical University.

### Induction of remote ischemic preconditioning (RIPC)

RIPC was induced in the fixed left hindlimb of rats using a modified blood pressure cuff. The cuff was inflated to 180–200 mmHg to block the blood flow through the left hindlimb femoral artery for 5 min (ischemia) followed by a 5-min deflation (reperfusion). Interruption of the blood supply to the hindlimb was confirmed by the disappearance of the pulse wave, a reduced body temperature in the limb, and cyanosis of the skin on the limb. The RIPC protocol consisted of 4 cycles per day of 5 min of ischemia followed by 5 min of reperfusion for 3 days before middle cerebral artery occlusion and reperfusion.

### Middle cerebral artery occlusion and reperfusion (MCAO/R) model

Rats were fasted for 12 h before surgery but remained free to drink water during that time. All rats were anesthetized by intraperitoneal injection of 10% chloral hydrate (350 mg/kg). MCAO/R surgery was conducted as described previously, with slight modification [19]. Briefly, the right common carotid artery (CCA), external carotid artery (ECA), internal carotid artery (ICA), and junction were carefully exposed and isolated through a midline neck incision under aseptic conditions. Silicone-coated sutures (head diameter 0.36 ± 0.02 mm, Beijing Sunbio Biotech Co., Ltd., Beijing, China) were inserted into the external carotid incision and passed through the ICA to the beginning of the middle cerebral artery to block its blood flow for 1 h followed by 24 h of reperfusion. The ipsilateral cerebral blood flow was monitored by laser Doppler flowmetry. The surgery was considered successful as long as the cerebral blood flow decreased by ≥ 70% from baseline. During the operation, rectal temperature was continuously monitored and maintained at 37 ± 0.5 °C with thermostatic surgery pad. After surgery, all rats were placed on warm blankets until recovery.

### Intracerebroventricular drug administration

Intracerebroventricular injection of the γ-secretase inhibitor DAPT or its vehicle, dimethyl sulfoxide (DMSO), was carried out as described previously [20, 21] Briefly, the rats were fixed in a stereotaxic apparatus (C type, ZH-Lanxing, Lihua Electronic Technology Development Co., Ltd., Xuzhou, China) after being anesthetized with 10% chloral hydrate (350 mg/kg, intraperitoneal injection). The coordinates of the right lateral ventricle were 0.8 mm posterior to the bregma, 1.5 mm from the midline, and 4.0 mm beneath the skull surface. Intracerebroventricular injections were performed using a Hamilton syringe attached to a 30-gauge needle 30 min prior to the MCAO surgery. DAPT (Cell Signaling Technology, Beverly, MA, USA; 50 μg/3 μl/rat, dissolved in 90% DMSO) or vehicle (3 μl, 90% DMSO) was administered intracerebroventricularly at a rate of 1 μl/min. After injection, the needle was kept in place for 5 min and then pulled out slowly. The craniotomies were closed with sterile bone wax, and the skin was sutured with silk thread after being disinfected with 75% ethanol.

### Assessment of neurological deficits

Neurological deficits were assessed after 24 h of reperfusion by an investigator who did not know the experimental groupings. The assessment was carried out using a 6-item scale with 4 levels for each item, based mainly on Garcia’s scale [22].

### Quantification of brain infarct volume

After the evaluation of neurological deficits, the rats were sacrificed under deep anesthesia. The brains were harvested and sectioned into 2-mm-thick coronal slices using a rat brain slice mold (Beijing Sunbio Biotech Co., Ltd., Beijing, China). The brain slices were stained with 1% 2,3,5-triphenyl-tetrazolium chloride solution (TTC, Sigma-Aldrich, St. Louis, MO, USA) in a dark, thermostatically controlled water bath at 37 °C for 15 min with agitation every 5 min. After being fixed with 4% paraformaldehyde solution for 24 h, the brain slices were scanned to obtain images, and the brain infarct area on each slice was calculated using ImageJ software. The infarct volume (%) for the brain was calculated with the following formula: (the volume of the contralateral hemisphere—the volume of the nonlesioned ipsilateral hemisphere)/(the volume of the contralateral hemisphere × 2) [23, 24].

### Primary culture and lentiviral transfection of hippocampal neurons

Primary hippocampal neurons were prepared from the hippocampi of Sprague Dawley rat embryos at 17 days of gestation using a previously described protocol [25]. Briefly, embryonic hippocampal tissues were gently minced into pieces using a sterile scalpel and incubated with papain solution at 37 °C for 30 min, followed by DNAzyme I for 3 min. DMEM/10% FBS was used to stop the enzymatic reaction. The cells were seeded onto Poly-l-lysine-coated plastic 6-well culture plates at a seeding density of 1–2× 10^5^/cm^2^ per well. After 4 h, the plating medium (DMEM/F12 + 2% B-27 + 2% FBS) was replaced with Neurobasal feeding medium (Neurobasal A + 2%B-27 + 1% l-glutamine). The neurons were fed every 3 days by removing half of the old media and replacing it with the same volume of fresh Neurobasal feeding media. After 7 days of culture, the neurons were labeled with an immunofluorescent MAP-2 antibody and prepared for follow-up experiments. A lentivirus containing the sh-Notch1 plasmid was used to transfect hippocampal neurons to interfere with the expression of the Notch gene. Lentiviral interference efficiency was measured using qRT-PCR and Western blotting 3 days after transfection.

### Oxygen-glucose deprivation (OGD) pretreatment and oxygen-glucose deprivation and reoxygenation (OGD/R) model construction

For preconditioning in vitro, hippocampal neurons were subjected to transient sublethal oxygen-glucose deprivation (OGD) according to previously described protocols [26, 27]. Briefly, hippocampal cultures were incubated in deoxygenated Neurobasal medium without glucose (Invitrogen, Carlsbad, CA, USA) under anoxic conditions (85% N_2_, 10% H_2_, 5% CO_2_) at 37 °C in an anaerobic incubator for 30 min. Then, the hippocampal cultures were cultured in the original Neurobasal medium and replaced in a normoxic incubator for 24 h. For the oxygen-glucose deprivation and reoxygenation (OGD/R) model, hippocampal cultures were exposed to OGD again for 3 h followed by a 24-h recovery in the original Neurobasal medium in a normoxic incubator (5% CO_2_ and 95% air).

### Cell viability assay

The viability of hippocampal neurons was quantified using a Cell Counting Kit-8 (CCK-8, Beijing Sunbio Biotech Co., Ltd., Beijing, China) according to the manufacturer’s protocol. Briefly, neurons were seeded onto a 96-well plate at a density of 2000 cell per well, with 6 replicate wells per group. The CCK-8 reaction solution was directly added to the medium in a volume of 10 μl per well. Finally, the number of viable cells was estimated by measuring *the optical density (OD) at 450 nm using a multidetection microplate reader (Molecular Devices, Sunnyvale, CA) after 4 h of incubation at 37* °C.

### Apoptosis detection by flow cytometry

Neuronal apoptosis was detected using fluorescence-activated cell sorting (FACS) with Annexin V-FITC/PI staining. Briefly, hippocampal neurons were harvested and resuspended in a binding buffer at a cell concentration of approximately 1 × 10 ^6^/ml after OGD/R or other treatments in different groups. An Annexin V-FITC Apoptosis Detection Kit (Keygen Biotech, Jiangsu, China) was used following the manufacturer’s protocol. Annexin V-FITC (1 μl) and propidium iodide (2 μl) were added to each 100 μl of cell suspension. After reacting for 15 min at room temperature in a dark environment, apoptotic cells were detected by FACS (BD Calibur; BD Biosciences, San Jose, CA, USA).

### Immunofluorescent staining

TUNEL and NeuN double immunofluorescence staining were used to detect neuronal apoptosis in the brain hippocampus area of the rats. For double immunofluorescence labeling, an Apoptosis Assay Kit (TUNEL, Beijing Sunbio Biotech Co., Ltd., Beijing, China) and anti-NeuN monoclonal antibody (Cell Signaling Technology, Beverly, MA, USA) were used as described [28]. In brief, brain sections measuring 40 μm in thickness were incubated with anti-NeuN primary antibodies (1:200 dilution) at 4 °C overnight, followed by a 1-h incubation with an immunofluorescently labeled secondary antibody (1: 500 dilution) at room temperature before TUNEL labeling. For TUNEL labeling, sections were incubated with a reaction mixture containing terminal deoxynucleotidyl transferase (TdT) and fluorescein-conjugated deoxyuridine triphosphate (dUTP) for 2 h at 37 °C. The nuclei were stained for 5 min with Hoechst stain at room temperature. All immunofluorescent labeling was detected using an automatic fluorescence microscope (OLYMPUS DP80; Olympus Co., Tokyo, Japan). Three sections per rat were examined, and the apoptotic neurons were counted in the images using the program ImageJ.

### Quantitative real-time polymerase chain reaction (qRT-PCR)

The expression level of Notch1 mRNA was detected by qRT-PCR. Briefly, total RNA was extracted by Trizol (Invitrogen, USA) after removing the genomic DNA using DNase I (Promega, USA) following the manufacturer’s instructions. The total RNA was reverse-transcribed to cDNA using a Reverse Transcription System (Promega, USA). Quantitative real-time PCR was performed on ABI Prism 7500 (Applied Biosystems) using SYBR® Green qPCR SuperMix-UDG (Invitrogen, USA). The cycling conditions were as follows: 2 min at 50 °C and then 2 min at 95 °C followed by 40 cycles of 15 s at 95 °C and 30 s at 60 °C. The PCR results were quantified using the threshold cycle (Ct) method.

### Western blot analysis

Protein expression was assessed by Western blotting. Briefly the ischemic penumbra tissues were collected according to a previously described method [29]. Proteins were extracted using a Total Protein Extraction Kit (Sangon Biotech, Shanghai, China), and the concentration was determined using a BCA protein assay kit (Nanjing Keygen Biotech, Jiangsu, China) according to the manufacturer’s protocol. The total protein was separated by gel electrophoresis (7.5–12.5% SDS-PAGE) and transferred to polyvinylidene fluoride (PVDF) membranes. The membranes were blocked in 5% fat-free milk powder for 1 h at room temperature, followed by an overnight incubation at 4 °C with rabbit anti-rat primary antibodies against NICD (1:1000 dilution, Cell Signaling Technology), Hes1 (1:1000 dilution, Cell Signaling Technology), IKKβ (1:1000 dilution, Abcam), Phospho-IKKα/β (1:1000 dilution, Cell Signaling Technology), Phospho-NF-κB p65 (1:1000 dilution, Cell Signaling Technology), NF-κB p65 (1:1000 dilution, Cell Signaling Technology), Bcl-2 (1:1000 dilution, Cell Signaling Technology), Bax (1:1000 dilution, Cell Signaling Technology), and β-actin (1:1000 dilution, Abcam). The membranes were washed four times in PBST (0.1 M PBS containing 0.3% Triton X-100), followed by 1 h of incubation with horseradish peroxide-conjugated anti-rabbit IgG secondary antibody at room temperature. All experiments were carried out at least three times. Chemiluminescence images were acquired using darkroom imaging techniques, and the relative quantity of proteins was analyzed using ImageJ and normalized to that of loading controls.

### Statistical analysis

Data were expressed as means ± SEMs. Neurological scores were presented as the median and interquartile range and analyzed using the Kruskal-Wallis H test. When the Kruskal-Wallis H test showed a significant difference, the Mann-Whitney *U* test with the Bonferroni correction was applied. All other data were analyzed using one-way ANOVA followed by the least significant difference (LSD) or Bonferroni’s method to evaluate the differences between groups if the variance was homogeneous, otherwise, the Games-Howell test was used. *P* < 0.05 was considered statistically significant. Statistical analysis was performed using the statistical program SPSS 23.0 (SPSS, IBM, Chicago, IL, USA).

## Results

### Effect of RIPC on neurological deficit scores and infarct volume in rats after MCAO/R

**Fig. 1:**
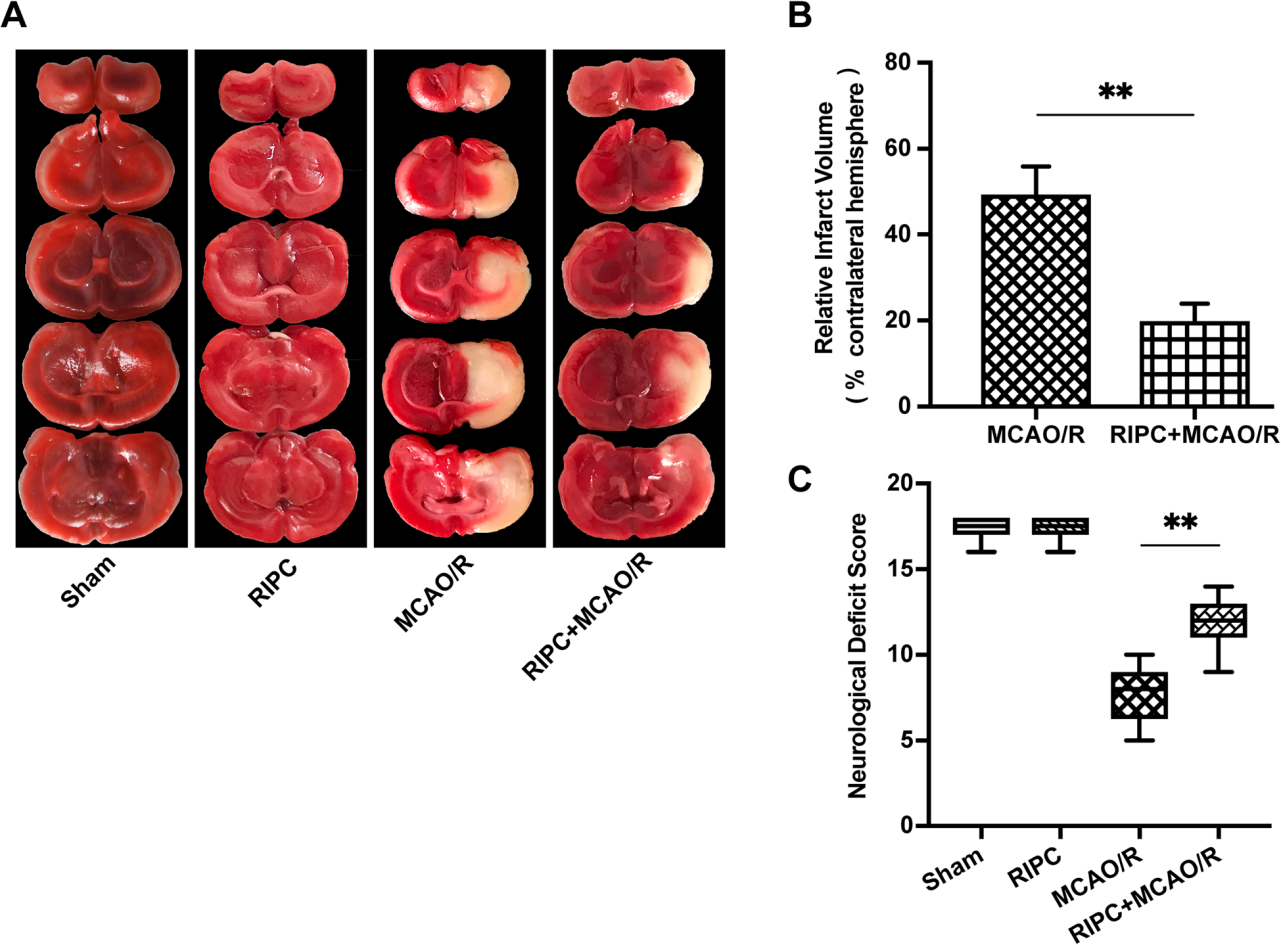
Effect of RIPC on neurological deficit scores and infarct volume in rats after MCAO/R. The neurological deficit scores and brain infarction volume were evaluated 24 h after reperfusion in rats in the four groups. **a** Cerebral infarct images (white, infarct tissue; red, noninfarct tissue) stained using 2,3,5-triphenyltetrazolium chloride in coronal sections of rat brains. **b** Brain infarct volume presented as a percentage of the intact hemisphere. Data are presented as the means ± SEM. **P < 0.01 vs. the MCAO/R group. **c** Neurological scores are presented as the median (interquartile range). **P < 0.01 vs. the MCAO/R group

Rats subjected to MCAO/R injury showed a significant motor behavioral deficit in the contralateral limbs. Neurological deficit scores were significantly decreased in the MCAO/R group. RIPC significantly increased neurological scores in comparison with those of the MCAO/R group (*P* < 0.01, Fig. 1c), suggesting a neuroprotective effect of RIPC in rats after MCAO/R.

To evaluate brain infarct volume, we used TTC staining 24 h after reperfusion. The infarct volume was reduced by 40.2% in the RIPC + MCAO/R group compared with the MCAO/R group (*P* < 0.01, Fig. 1b)*.* No infarction or edema formation was observed in either the sham group or the RIPC group (Fig. 1a).

### Effects of RIPC on the expression of NICD, Hes1, IKKβ, and NF-κB p65 in the ischemic penumbra after MCAO/R

**Fig. 2:**
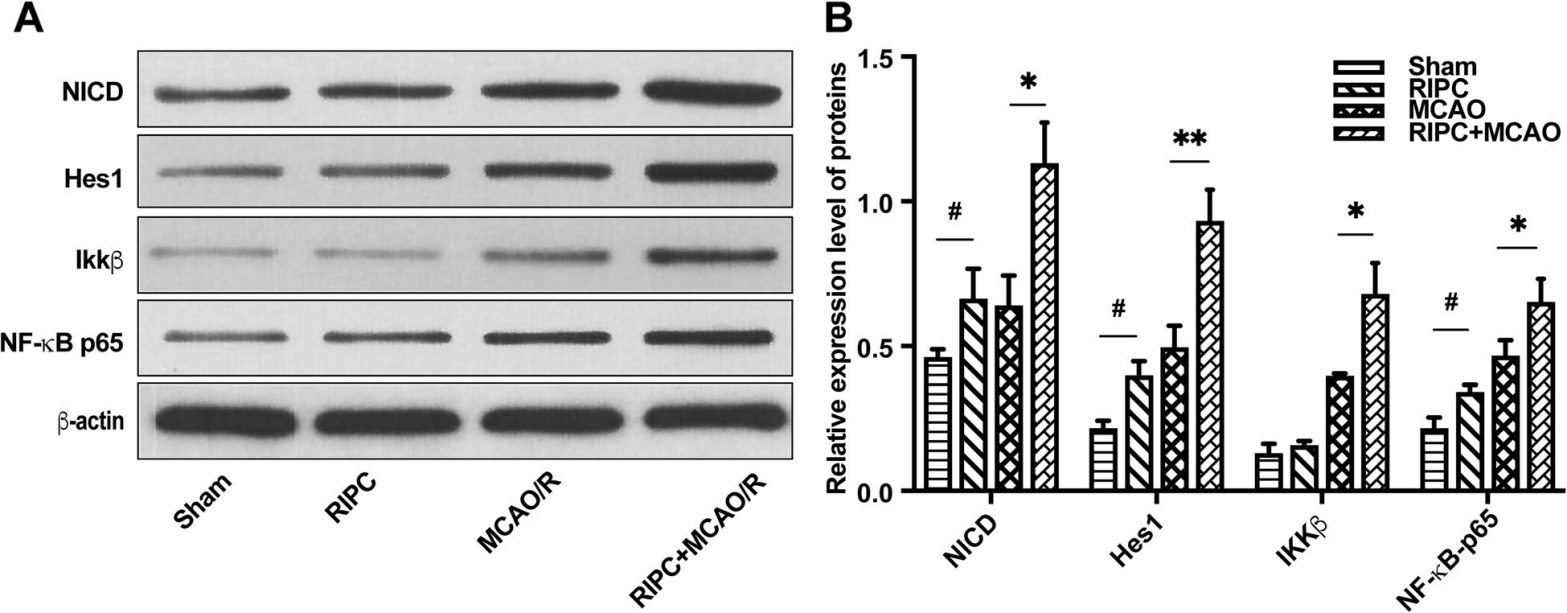
RIPC activated the Notch and NF-κB signaling pathways in the ischemic penumbra after MCAO/R. **a** Protein bands of NICD, Hes1, IKKβ, NF-κB p65, and β-actin from Western blot analysis. **b** RIPC significantly upregulated the expression of proteins related to the Notch and NF-κB signaling pathways. Data are presented as the means ± SEM. *P < 0.05, **P < 0.01, vs. the MCAO/R group; ^**#**^*P* < 0.05 vs. the sham group

To explore the effects of RIPC on the Notch and NF-κB signaling pathways in the brain after MCAO/R, we conducted Western blots to investigate the expression of NICD, Hes1, IKKβ, and NF-κB p65 in the ischemic penumbra after 24 h of reperfusion. The RIPC group had higher expression of NICD, Hes1, and NF-κB p65 than the sham group. In comparison with the MCAO/R group, RIPC significantly upregulated the expression of NICD, Hes1, IKKβ, and NF-κB p65 in the RIPC + MCAO/R group, suggesting that RIPC plays a role in activating the Notch and NF-κB signaling pathways in the brain after MCAO/R (Fig. 2a, b).

### Effects of OGD preconditioning on the levels of cell viability and cell apoptosis in primary hippocampal neurons after OGD/R injury

**Fig. 3:**
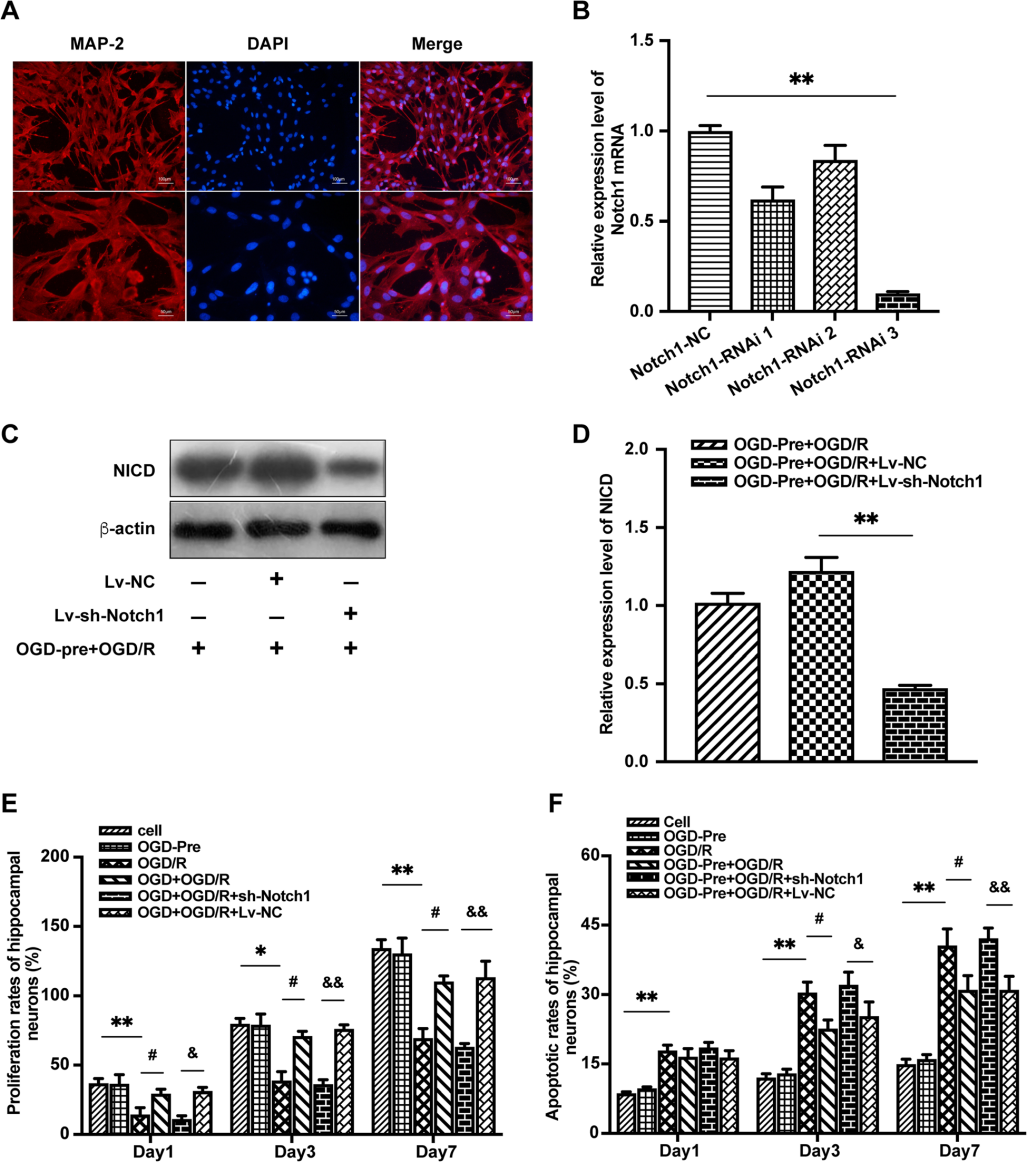
OGD preconditioning-induced neuroprotection was attenuated by Notch1-RNAi in vitro. **a** Hippocampal neurons were identified by Map-2 antibody labeling (red), and the purity was over 95%. **b** Screening results of Notch1 RNA interference fragments. **P < 0.01 vs. Notch1-NC group. **c**, **d** The expression of NICD was significantly reduced by Lv-sh-Notch1. **P < 0.01 vs. Lv-NC group. **e**, **f** OGD preconditioning significantly improved hippocampal neuronal proliferation rates and reduced neuronal apoptosis after OGD/R. The neuroprotective effect was attenuated by Notch1-RNAi. Data are presented as the means ± SEM, *P < 0.05, **P < 0.01 vs. the control cell group; ^**#**^*P* < 0.05 vs. OGD/R group. ^&^*P* < 0.05, ^&&^*P* < 0.01 vs. OGD-Pre + OGD/R + Lv-NC group

Hippocampal neurons were identified by immunofluorescent MAP-2 antibody (Fig. 3a). Notch1 RNA interference efficiency was identified by qRT-PCR and Western blotting (Fig. 3b, c, d). Neuronal viability was assessed using the CCK-8 assay. After primary hippocampal neurons were subjected to 3 h of OGD followed by 24 h of recovery, cell viability was significantly decreased compared with that of the control cell group on days 1, 3, and 7 after OGD/R (Fig. 3e). By contrast, cell viability was not affected by 30 min of OGD preconditioning. Furthermore, OGD preconditioning significantly increased cell viability after OGD/R injury on day 3 and day 7 after OGD/R, but the neuroprotective effect was attenuated by Notch1-RNAi (*P* < 0.05, Fig. 3e).

To determine the effects of OGD preconditioning on OGD/R-induced apoptosis of hippocampal neurons, we used FACS with Annexin V-FITC/PI staining. The results showed that neuronal apoptosis was significantly increased after OGD/R injury. No significant neuronal apoptosis was observed after OGD preconditioning compared with the control cell group. OGD preconditioning significantly reduced neuronal apoptosis on day 3 and day 7 after OGD/R (*P* < 0.05, Fig. 3f). However, the neuroprotective effect was attenuated by Notch1-RNAi, suggesting an important role of the Notch1 signaling pathway in the neuroprotection provided by OGD preconditioning (Fig. 3f).

### Effects of DAPT on MCAO/R injury and Notch1-RNAi on OGD/R injury in vivo and in vitro, respectively

**Fig. 4:**
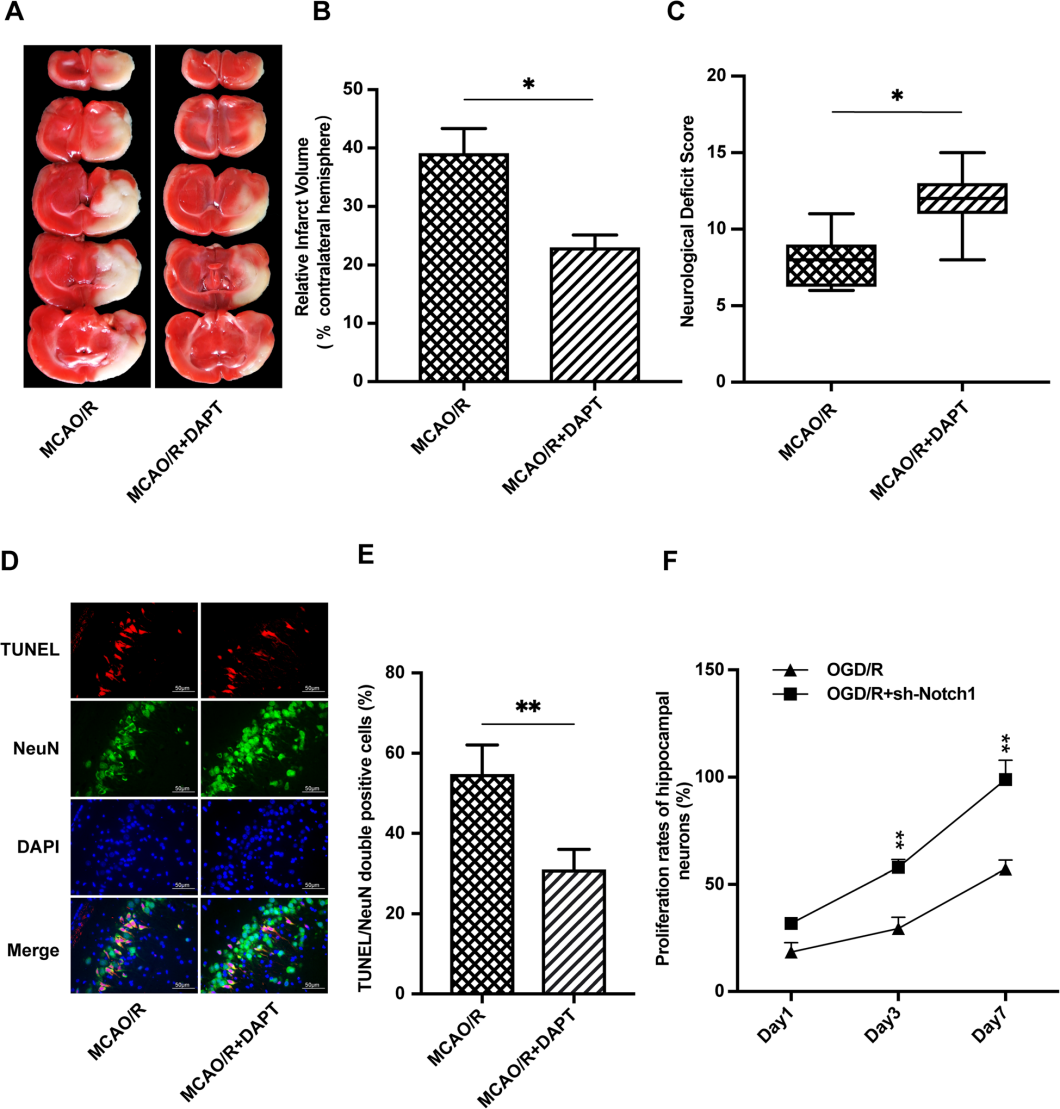
DAPT and Notch1-RNAi alleviated MCAO/R and OGD/R injury in vivo and in vitro, respectively. **a**, **b** Brain infarct volume presented as a percentage of the intact hemisphere. *P < 0.05 vs. the MCAO/R group. **c** Neurological scores are presented as the median (interquartile range), *P < 0.05 vs. the MCAO/R group. **d**, **e** Neuronal apoptosis assayed by TUNEL, NeuN, and DAPI immunofluorescence staining. The percent of TUNEL and NeuN double-positive cells. **P < 0.01 vs. the MCAO/R group. **f** Notch1-RNAi significantly improved hippocampal neuronal proliferation rates on day 3 and day 7 after OGD/R injury. **P < 0.01 vs. the OGD/R group

**Fig. 5:**
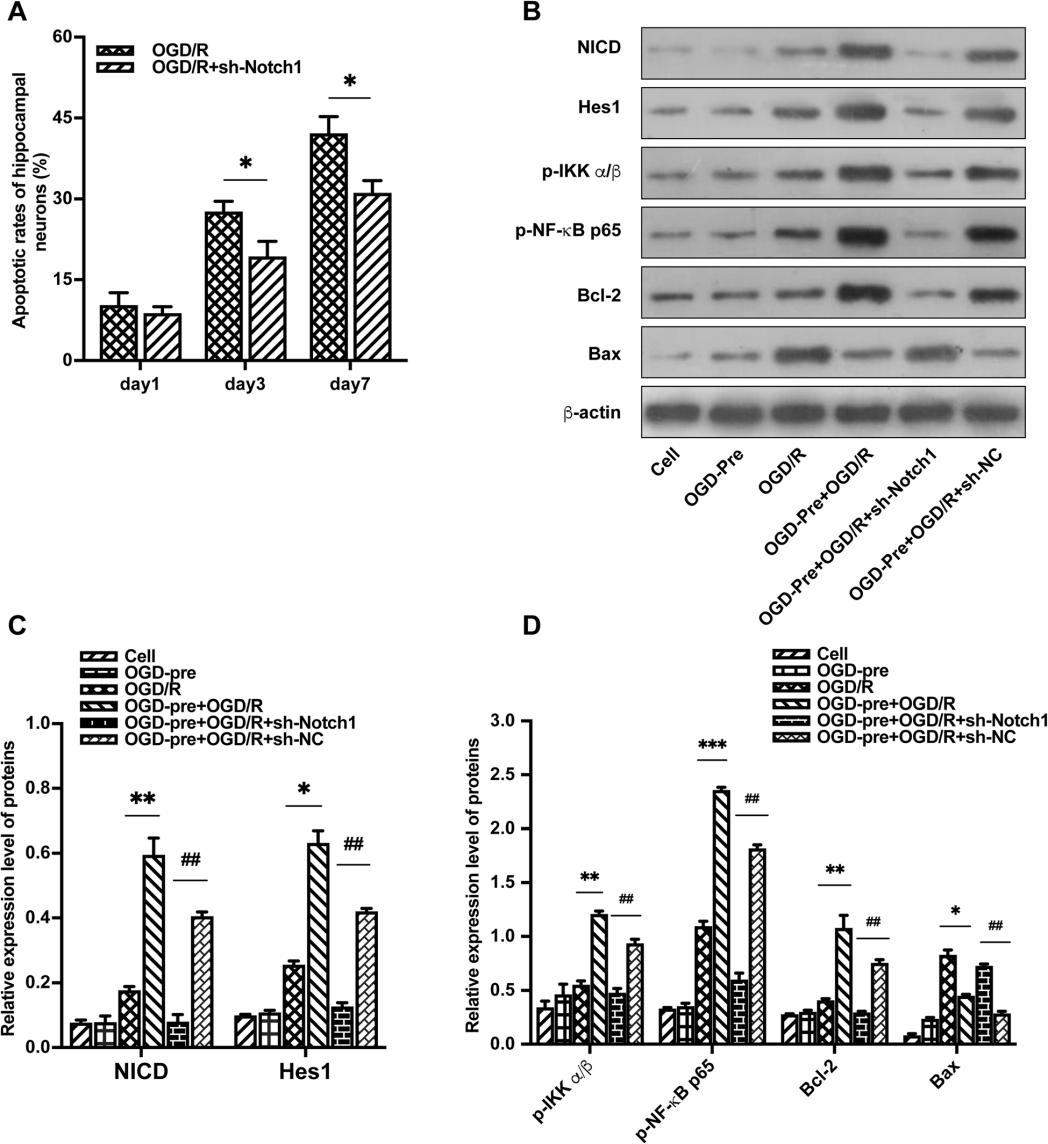
In the hippocampal neuronal OGD/R model, OGD-preconditioning activated the Notch1 and NF-κB signaling pathways, and the NF-κB signaling pathway was suppressed by Notch1-RNAi. **a** Notch1-RNAi significantly reduced neuronal apoptosis on day 3 and day 7 following direct lethal OGD/R injury in vitro, *P < 0.05 vs. OGD/R group. **b** Protein bands of NICD, Hes1, p-IKK α/β, p-NF-κB p65, Bcl-2, Bax, and β-actin from Western blot analysis. **c**, **d** OGD-preconditioning significantly upregulated the expression of proteins related to the Notch1 and NF-κB signaling pathways after OGD/R. The expression of p-IKK α/β and p-NF-κB p65 was suppressed by Notch1-RNAi. Data are presented as the means ± SEM in **c** and **d**; *P < 0.05, **P < 0.01, ***P < 0.001 vs. the OGD/R group; ^**##**^*P* < 0.01 vs. OGD-Pre + OGD/R + sh-NC group

DAPT significantly increased neurological scores and reduced infarct volume and neuronal apoptosis after direct lethal MCAO/R injury in vivo (Fig. 4a–e). Notch1-RNAi significantly increased hippocampal neuronal cell viability and reduced neuronal apoptosis on day 3 and day 7 following direct lethal OGD/R injury in vitro (Fig. 4f and Fig. 5a).

### Effects of OGD preconditioning on the expression of NICD, Hes1, p-IKK α/β, p-NF-κB p65, Bcl-2, and Bax in primary hippocampal neurons after OGD/R

To determine the effect of OGD preconditioning on the Notch and NF-κB signaling pathways in primary hippocampal neurons after OGD/R, we used Western blot analysis to examine the expression of NICD, Hes1, p-IKK α/β, p-NF-κB p65, Bcl-2, and Bax in primary hippocampal neurons after OGD/R. The OGD-PC group had significantly higher expression of NICD, Hes1, p-IKK α/β, and p-NF-κB p65 than the control cell group. In comparison with the OGD/R group, OGD preconditioning significantly upregulated the expression of NICD, Hes1, p-IKK α/β, p-NF-κB p65, and Bcl-2 in the OGD + OGD/R group, suggesting that OGD preconditioning plays a role in activating the Notch and NF-κB signaling pathways in primary hippocampal neurons. However, the expression of Bax decreased in the OGD + OGD/R group compared to the OGD/R group. The expression of p-IKK α/β and p-NF-κB p65 was suppressed by Notch1-RNAi, suggesting that the NF-κB signaling pathway was regulated by the Notch1 signaling pathway in primary hippocampal neurons after OGD/R (Fig. 5b–d).

### RIPC-induced neuroprotection was attenuated by the Notch1 signaling pathway inhibitor DAPT in vivo

**Fig. 6:**
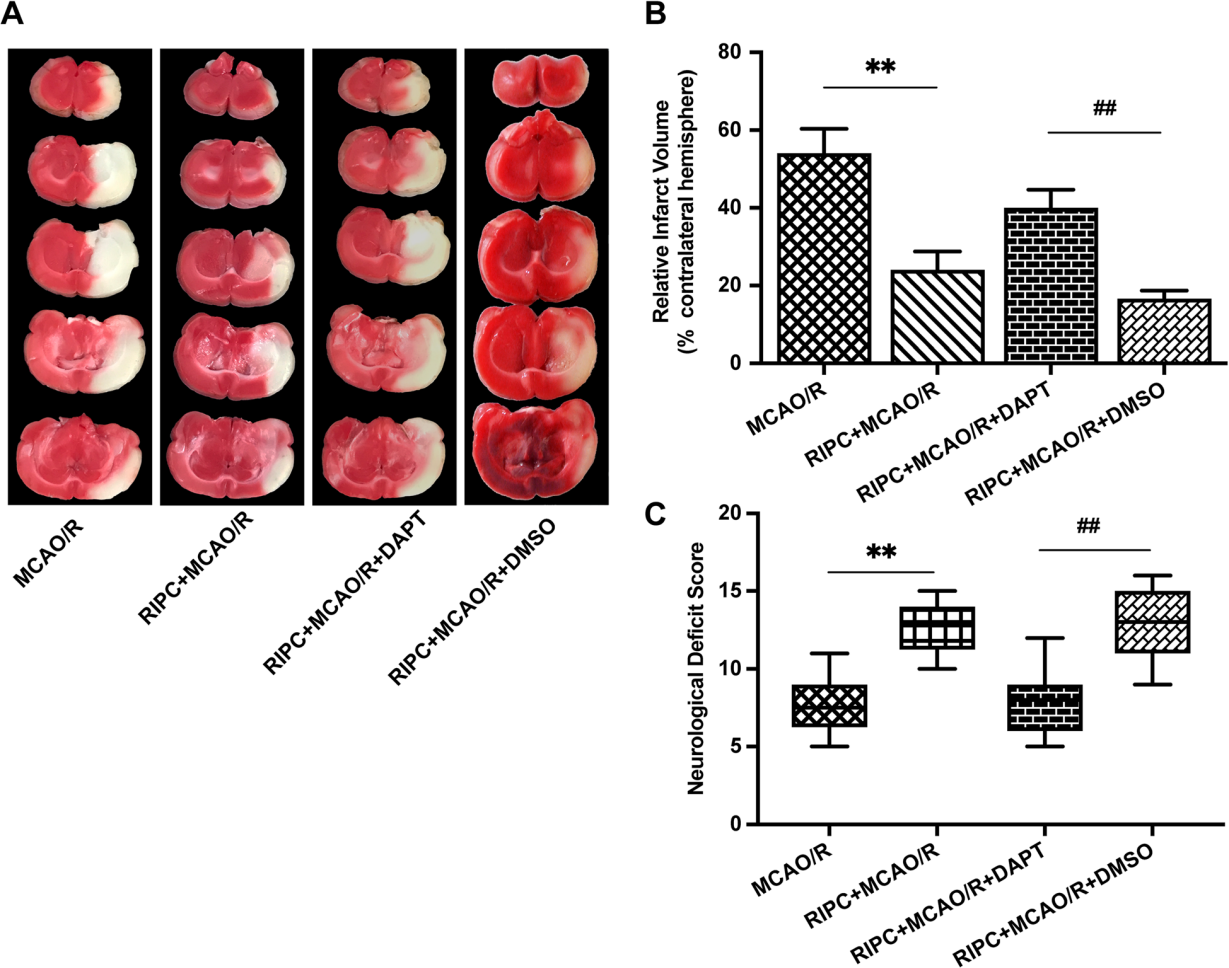
RIPC-induced neuroprotection was attenuated by a Notch1 signaling pathway inhibitor in vivo. The neurological deficit scores and brain infarction volume were evaluated 24 h after reperfusion in rats in the 4 groups. **a** Images of focal cerebral infarction staining by TTC in coronal brain slices (white, infarct tissue; red, noninfarct tissue). **b** Brain infarct volume presented as a percentage of the intact hemisphere. Data are presented as the means ± SEM. **P < 0.01 vs. MCAO/R group, ^**##**^*P* < 0.01 vs. the RIPC + MCAO/R + DAPT group. **c** Neurological deficit scores are presented as the median with interquartile range. **P < 0.01 vs. MCAO/R group, ^**##**^*P* < 0.01 vs. the RIPC + MCAO/R + DAPT group

To further confirm whether RIPC-induced neuroprotection is mediated by the Notch1 signal pathway, we administered the Notch1 pathway inhibitor DAPT to the rats by the intracerebroventricular route and evaluated by neurological deficit scores and relative brain infarct volume. Neurological deficit scores were significantly higher in the RIPC + MCAO/R group than in the MCAO/R group. However, the neurological deficit scores significantly decreased when the Notch1 signaling pathway was inhibited by DAPT (*P* < 0.01, Fig. 6c). RIPC significantly decreased the relative brain infarct volume in comparison with that of the MCAO/R group. However, the neuroprotective effect was reversed by DAPT. These results indicated that Notch1 signaling activation contributes to the neuroprotection provided by RIPC (*P* < 0.01, Fig. 6a, b).

### RIPC significantly reduced hippocampal neuronal cell apoptosis in rats after MCAO/R, but the neuroprotective effects were attenuated by an inhibitor of the Notch1 signaling pathway

**Fig. 7:**
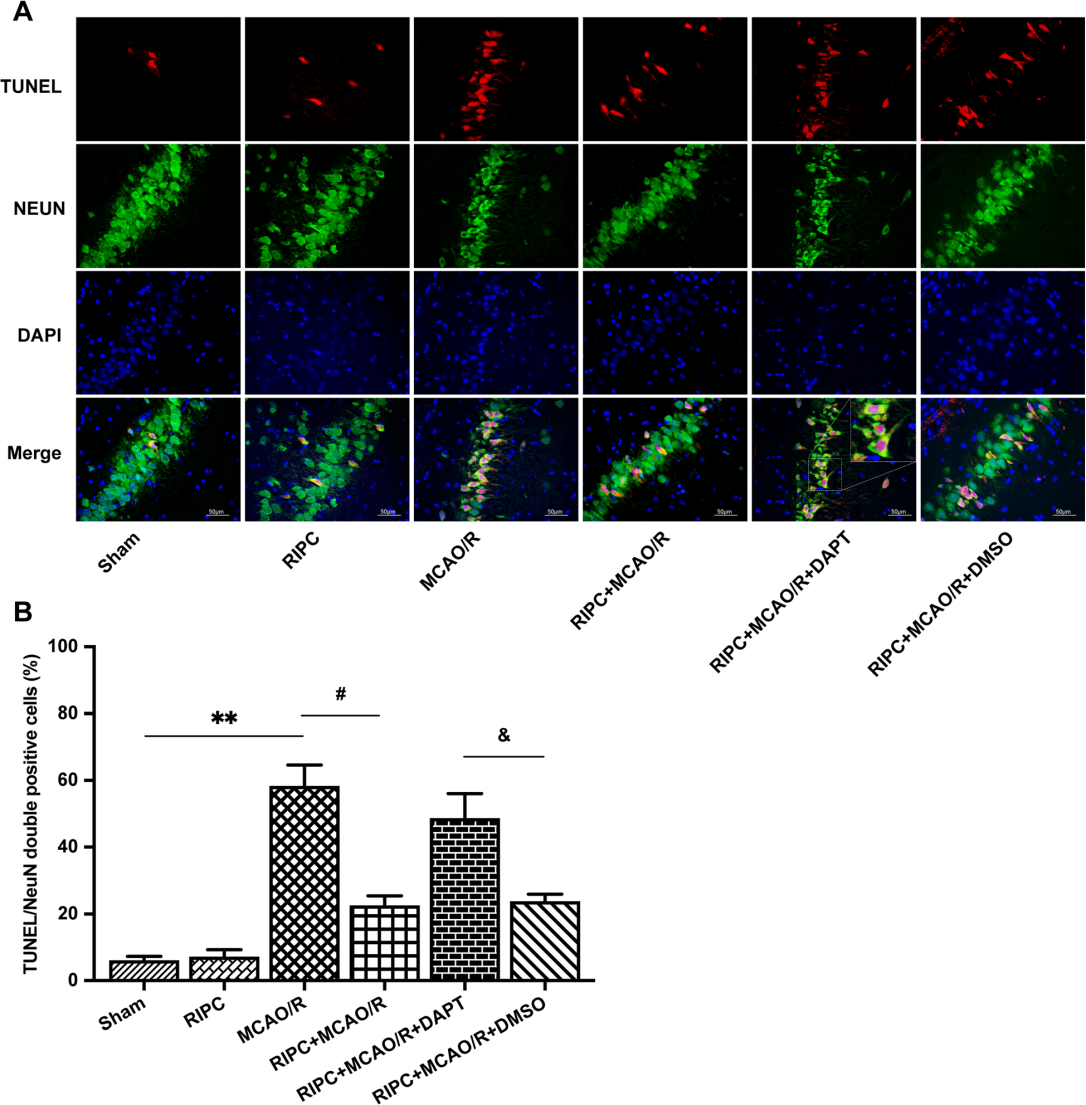
RIPC significantly reduced hippocampal neuronal cell apoptosis in rats after MCAO/R. However, the neuroprotective effects were attenuated by an inhibitor of the Notch1 signaling pathway. **a** Apoptosis was assayed by TUNEL, NeuN, and DAPI immunofluorescence staining. **b** The percent of TUNEL and NeuN double-positive cells. **P < 0.01 vs. the Sham group, ^**#**^*P* < 0.05 vs. the MCAO/R group, ^&^*P* < 0.05 vs. the RIPC + MCAO/R + DMSO group

A NeuN/TUNEL assay was used to elucidate the effect of RIPC on neuronal apoptosis in the rat hippocampus after MCAO/R. TUNEL and NeuN double immunofluorescence staining represents the rate of apoptosis. We found that MCAO/R injury increased the number of NeuN/TUNEL-positive cells in the hippocampus area, while RIPC significantly downregulated the proportion of NeuN/TUNEL-positive cells compared with that of the MCAO/R group. However, the antiapoptotic effect was attenuated by DAPT (*P* < 0.01, Fig. 7a, b). These results illustrate that RIPC significantly reduced hippocampal neuronal apoptosis in rats after MCAO/R. However, the neuroprotective effects were attenuated by an inhibitor of the Notch signaling pathway.

### The NF-κB signaling pathway is activated by RIPC but attenuated by the Notch1 pathway inhibitor DAPT

**Fig. 8:**
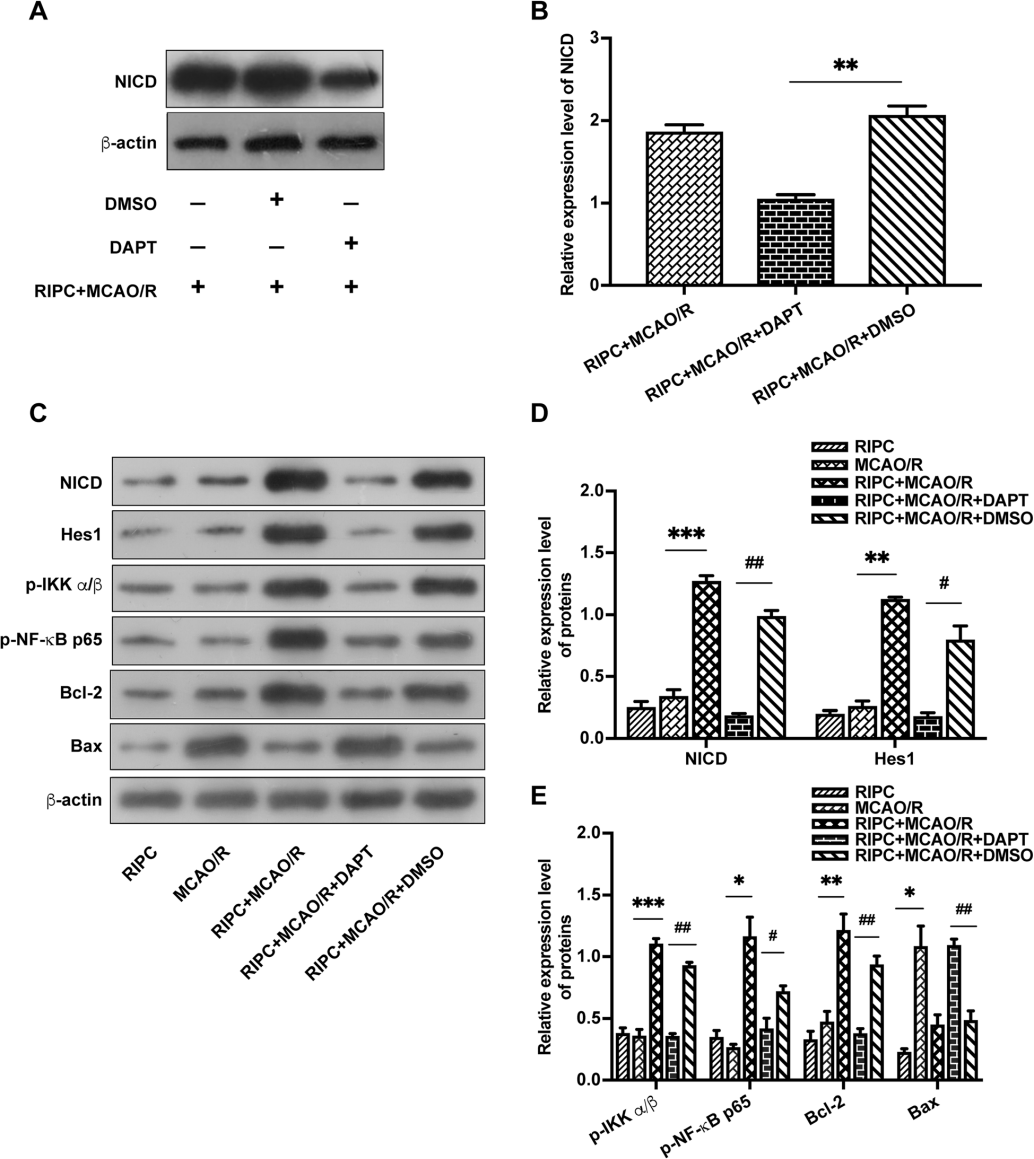
RIPC activated the Notch1 and NF-κB signaling pathways in rats after MCAO/R. NF-ΚB signaling pathway activity was suppressed by the Notch1 signaling pathway inhibitor DAPT. **a**, **b** DAPT significantly decreased the expression of NICD in rats after RIPC + MCAO/R. **P < 0.01 vs. DMSO group. **c** Protein bands of NICD, Hes1, p-IKK α/β, p-NF-κB p65, Bcl-2, Bax, and β-actin from Western blot analysis. **d**, **e** RIPC significantly upregulated the expression of proteins related to the Notch1 and NF-κB signaling pathways. The Notch1 signaling pathway inhibitor DAPT reduced the activity of the NF-κB signaling pathway. Data are presented as the means ± SEM; *P < 0.05, **P < 0.01, ***P < 0.001 vs. the RIPC + MCAO/R group, ^**#**^*P* < 0.05, ^**##**^*P* < 0.01 vs. the RIPC + MCAO/R + DMSO group

To determine whether Notch1 modulates the activity of the NF-κB signaling pathway during RIPC, we measured the expression of p-IKK α/β, p-NF-κB p65, Bcl-2, and Bax in the ischemic penumbra of rats that received DAPT compared to rats that did not. RIPC significantly upregulated the expression of NICD, Hes1, p-IKK α/β, p-NF-κB p65, and Bcl-2 but suppressed the expression of Bax. However, DAPT markedly suppressed the RIPC-induced upregulation of NICD and Hes1 and attenuated the expression of p-IKK α/β, p-NF-κB p65, and Bcl-2 (Fig. 8c–e). These results suggested that RIPC activated the Notch and NF-κB signaling pathways in rats after MCAO/R. NF-κB pathway activity was suppressed by the Notch1 pathway inhibitor DAPT in vivo.

## Discussion

The aim of this study was to investigate the underlying mechanism of RIPC-mediated neuroprotection against cerebral ischemia-reperfusion injury and the roles of the Notch1 and NF-κB signaling pathways in RIPC-induced cerebral ischemic tolerance. Solid evidence from diverse preclinical models and species and clinical trials has demonstrated that limb RIPC can effectively alleviate cerebral ischemia-reperfusion injury [30–32]. However, the underlying mechanisms of RIPC-mediated neuroprotection are not fully understood [33, 34]. The present study demonstrates that RIPC significantly improved the outcome of rats subjected to MCAO/R and that the neuroprotective effect of RIPC is related to activation of the Notch1 and NF-κB signaling pathways. In this study, we found that RIPC significantly reduced brain infarct volume and hippocampal neuronal apoptosis while improving neurological deficit scores in rats after MCAO/R, but the neuroprotective effect was attenuated by blocking the Notch1 pathway in vitro and in vivo. Furthermore, we found that the activity of the NF-κB pathway was affected by the Notch1 pathway during RIPC, exhibiting a synergistic change. These findings imply that the Notch1 signaling pathway is essential in the neuroprotective effects of RIPC, which may be achieved by modulating the activity of the NF-κB signaling pathway.

Limb RIPC is an attractive potential therapeutic strategy against ischemic stroke. RIPC-induced cerebral ischemic tolerance is achieved by intermittent, repeated, and sublethal episodes of ischemia and reperfusion in a limb, distant from the brain [33]. It has been reported that ischemic preconditioning-mediated ischemic tolerance is achieved in two different time windows: early or rapid preconditioning and delayed or classical preconditioning [35, 36]. The delayed tolerance is achieved due to genetic alterations and protein synthesis and is the more significant of the two phases because of its longer duration [37]. The time interval between preconditioning and the subsequent insult is critical [38]. It has been reported that when the interval between ischemic preconditioning and subsequent potentially lethal cerebral ischemia is less than 3 h. IPC has no protective effect but aggravates neuronal injury. However, when the interval is extended to 12–48 h, ischemic tolerance prevails in the brain [39, 40]. In previous studies, RIPC has been induced by 4 cycles of 5 min of left hind limb ischemia followed by 5 min of reperfusion for 1 day [41, 42]*.* It has been reported that repeated limb remote ischemic postconditioning provides cardioprotection against myocardial infarction more effectively than a single episode of limb preconditioning [43]. In our study, to durable, robust neuroprotection, we initiated 3-day RIPC before MCAO/R, with each day’s procedure including 4 cycles of 5 min of ischemia followed by 5 min of reperfusion in the left hindlimb. The results showed that ischemic tolerance induced by RIPC effectively alleviated ischemia-reperfusion injury in the rats after MCAO/R (Figs. 1, 6, and 7). OGD/R as a classical in vitro model for ischemia-reperfusion injury has been widely used in ischemic stroke studies [24, 44]. The results from our in vitro study show that OGD preconditioning provides potent neuroprotection in hippocampal neurons exposed to OGD/R by improving neuronal cell proliferation activity and antiapoptotic effects (Fig. 3e, f). This is consistent with previous observations [26, 45].

The underlying mechanisms of RIPC-mediated cerebral ischemic tolerance are intricate, multifactorial, and currently not well understood, although many preclinical studies and human clinical trials have been carried out. It has been reported that humoral and neurogenic pathways are involved in triggering intracellular signal pathways for neuroprotection [46–48]. The signaling pathway responsible for RIPC in the brain during ischemic injury is unclear. The involvement of multiple mediators suggests that more than one pathway is involved, which may act sequentially or in parallel for an additive effect [36]. Activation of Notch signal has been reported to play an important cardioprotective role following cardiac ischemic injury [49]. Notch signaling activation contributes to cardioprotection provided by ischemic preconditioning [9]. It was demonstrated that pretreatment with sevoflurane and isoflurane could reduce neuronal cell apoptosis and promote proliferation of neural stem cells during ischemia-reperfusion injury by activating the Notch signaling pathway. The neuroprotective effects of pretreatment were abrogated by blockade of the Notch signaling pathway, suggesting the critical role of Notch in the induction of cerebral ischemic tolerance [5, 8]. A recent study suggests that RIPC promotes arteriogenesis in the ischemic rat brain via increasing Notch signaling activity after ischemic stroke [10]. In our previous study, we also found that the neuroprotective effects of cerebral ischemic preconditioning (cIPC) in a rat MCAO/R model are mediated by the preactivation of the Notch1 signaling pathway [50]. In this study, we found that RIPC significantly increased the expression of NICD and Hes1 in the ischemic penumbra of the rats after MCAO/R (Fig. 2a, b; Fig. 8c, d), suggesting a stimulating effect of RIPC on Notch1 activity of the rats subjected to ischemia/reperfusion. We also found that Notch1 signaling in hippocampal neurons is activated by OGD preconditioning in the OGD/R model in vitro (Fig. 5b, c). In this study, we have shown that, 3 consecutive days of RIPC remarkably reduces ischemic infarct volume and hippocampal neuronal apoptosis in rats subjected to MCAO/R, but the neuroprotective effect of RIPC was attenuated by Notch1-RNAi in vitro and DAPT in vivo. Our results indicate that the Notch1 signaling pathway is essential for the formation of RIPC-mediated neuroprotective effects. Previous investigators suggested that Notch signaling might contribute to brain damage and functional outcome by activating microglial cells and stimulating the infiltration of proinflammatory leukocytes in ischemic stroke [4]. In our study, we found that when the Notch1 pathway was directly activated by lethal MCAO/R or OGD/R injury, it might play a damaging role, and blocking Notch1 signaling with DAPT or Notch1-RNAi could ameliorate the injury (Fig. 4 and Fig. 5a). The different observations may be related to the manner and the time point in which Notch signaling is activated, as well as its dynamically changing roles in different pathophysiological stages [8, 10, 50]. It was reported that cerebral ischemia and hypoxia could activate the Notch1 signaling pathway and four prominent interacting pathways (NF-κB, p53, HIF-1α and Pin1) that converge on a conserved DNA-associated nuclear multi-protein complex, which controls the expression of genes that could determine the fate of neurons. When neurons experience a sublethal ischemic stimulation (preconditioning), the nuclear multi-protein complex upregulates adaptive stress response genes encoding proteins that promote neuronal survival, but when ischemia is a lethal stimulation, the nuclear multi-protein complex induces genes encoding proteins that trigger and execute a neuronal death program [51]. In our study, we observed that when the Notch1 signaling was pre-activated by RIPC, it upregulated the expression of downstream protective genes by modulating the NF-κB pathway, resulting in neuroprotective effects against cerebral I/R injury. Previous studies have suggested that activation of NF-κB is essential in the induction of gene expression related to the neuroprotection mediated by ischemic preconditioning [52]. The activation of NF-κB in hippocampal neurons by ischemic preconditioning is a key event in brain ischemic tolerance [12]. In addition, it has been suggested that the activation of NF-κB in pyramidal neurons of the hippocampal CA1 region is an important axis of the IPC-mediated neuroprotective mechanism [11]. In the pathophysiological process of ischemia-reperfusion injury, the activation of NF-κB in different cell types determines its neuronal pro-death or prosurvival effects: NF-κB activation in neurons promotes their survival and plasticity, whereas NF-κB activation in glial cells enhances neuronal death [11, 53, 54]. In this study, we found that RIPC could upregulate the expression of p-IKK α/β and p-NF-κB p65 while activating the Notch1 signaling pathway, suggesting that RIPC activates the NF-κB pathway in cerebral ischemia-reperfusion injury. However, we also found that the activity of the NF-ΚB pathway was inhibited by the Notch1 inhibitors DAPT and Notch1-RNAi, suggesting that the NF-κB pathway is a downstream target of the Notch1 signaling pathway in RIPC. The mechanisms may be associated with reciprocal transcriptional regulation and physical interaction between Notch1 and NF-κB components, as well as the binding of Notch1 to components of the IKK complex [13, 55]. It is believed that NF-κB plays a critical role in the induction of neuroprotective antiapoptotic gene products, such as MnSOD and Bcl-2, which are known to contribute to ischemic tolerance [12]. In our study, we found that pre-activation of the NF-κB pathway in neuronal cells upregulated the expression of the downstream antiapoptotic gene Bcl-2, and reduced the expression of the proapoptotic gene Bax, producing neuroprotective effects (Fig. 5b, d; Fig. 8c, e). Unbalance in the expression of proapoptotic and antiapoptotic proteins may be related to the translocation of different subunits of the NF-kB pathway into the nucleus to activate different target genes [56]. Bcl-2 and Bax regulate the release of cytochrome C from the mitochondria via alteration of mitochondrial membrane potential and permeability, thereby regulating activation of caspases and the intrinsic mitochondrial apoptotic pathway. The proapoptotic activities of Bax are counteracted by Bcl-2 via retrotranslocating Bax from the mitochondria back into the cytosol [3, 57, 58].

Based on our in vitro and in vivo experiments, we can conclude that RIPC-mediated cerebral ischemic tolerance can be achieved by pre-activating the Notch1 pathway in neurons, thereby upregulating the activity of p-IKK α/β and activating the NF-κB pathway in neuronal cells, resulting in changes in the expression of the apoptosis-related downstream genes Bcl-2 and Bax.

## Conclusions

In summary, this study demonstrated that the neuroprotective effect of RIPC on cerebral I/R injury was associated with activation of the Notch1 and NF-κB pathways in neurons. The NF-κB pathway is a downstream target of the Notch1 signaling pathway in the protective effect of RIPC against focal cerebral I/R injury. Further studies are needed to clarify the exact mechanism of the interaction between the Notch1 and NF-κB pathways in RIPC-mediated neuroprotection. Since studies have now begun to translate RIPC to the clinic in several randomized trials with exciting outcomes, our results may provide some theoretical support for this clinical transformation.

## Data Availability

All data supporting the conclusions of this manuscript are provided in the text and figures.

## Abbreviations

CCK-8: Cell counting kit-8
DMSO: Dimethyl sulfoxide
FACS: Fluorescence-activated cell sorting
I/R: Ischemia-reperfusion
IKK: IκB kinase
IPC: Ischemic preconditioning
MCAO/R: Middle cerebral artery occlusion and reperfusion
OGD: Oxygen-glucose deprivation
OGD/R: Oxygen-glucose deprivation and reoxygenation
RIPC: Remote ischemic preconditioning
TTC: 2,3,5-Triphenyl-tetrazolium chloride solution
